# Chronic Inflammatory Demyelinating Polyneuropathy as the Initial Presentation of Systemic Lupus Erythematosus Successfully Treated With Cyclophosphamide

**DOI:** 10.7759/cureus.51648

**Published:** 2024-01-04

**Authors:** Andrés D Sastre Martínez, María J Tróchez Ortiz, Lizeth V Zuluaga Gómez, Christian D Messu Llano

**Affiliations:** 1 Internal Medicine, Universidad del Valle, Cali, COL; 2 Medicine, Faculty of Health Sciences, Universidad Icesi, Cali, COL; 3 Medicine, Unidad Central del Valle del Cauca, Tuluá, COL; 4 Internal Medicine, Hospital Universitario del Valle, Cali, COL

**Keywords:** systemic lupus erythematosus, inflammatory myositis, lupus nephritis, cyclophosphamide pulse, chronic inflammatory demyelinating polyneuropathy (cidp)

## Abstract

Systemic lupus erythematosus (SLE) is an autoimmune disorder that can manifest with a wide range of clinical features, including peripheral nervous system involvement. Among the neurological complications associated with SLE, chronic inflammatory demyelinating polyneuropathy (CIDP) is a rare but significant entity. This case report explores the complex relationship between CIDP and SLE, emphasizing the challenges in diagnosis and the complexities of treatment strategies. We present the case of a patient diagnosed with CIDP as the initial manifestation of SLE, who exhibited a remarkable response to a unique treatment approach. This case underscores the potential overlap of these two conditions, the need for individualized diagnostic, and the importance of considering lupus activity when making therapeutic decisions. While conventional treatment approaches for CIDP are established, the management of CIDP in the context of SLE requires a thorough approach. This report presents a case where early intervention with steroids and cyclophosphamide yielded favorable outcomes, providing insights into alternative treatment options. As this subset of patients remains underrepresented in clinical trials, further research is needed to establish clear guidelines for the management of CIDP in SLE, optimizing patient care while minimizing risks associated with immunomodulatory therapies.

## Introduction

Systemic lupus erythematosus (SLE) is a chronic connective tissue disease that affects multiple systems and organs. Neurological involvement is common, and the term "neuropsychiatric lupus" has been proposed to encompass the neurologic manifestations [1], including central and peripheral nervous system involvement, with neuropathies being one of the most common forms of presentation [2]. Chronic inflammatory demyelinating polyneuropathy (CIDP) is a clinical condition characterized by progressive weakness and sensory loss mediated by an immune response. It is possible that CIDP may be the initial clinical manifestation of lupus [3,4]. Initial treatment typically involves corticosteroids, intravenous immunoglobulin (IVIG), or plasma exchange. However, other immunomodulators such as rituximab, cyclosporine, or cyclophosphamide may be considered [5]. Here, we present the case of a patient with a CIDP diagnosis as the initial manifestation of SLE, who experienced satisfactory improvement following treatment with intravenous (IV) steroids and cyclophosphamide.

## Case presentation

A 32-year-old woman of African descent, with no relevant past medical history, presented to the emergency department due to two months of progressive leg weakness leading to walking difficulties. The patient also reported paresthesia in her fingers and the soles of her feet. Over time, the weakness and paresthesia extended to the upper limbs. She also complained about joint pain in her fingers, generalized hair loss, swelling in the lower limbs, and unintentional weight loss over the last six months.

Brain and cervical-thoracic-lumbar spinal cord magnetic resonance imaging ruled out central nervous system involvement. A lumbar puncture demonstrated albumin-cytologic dissociation, and central nervous system infections were excluded (Table 1).

**Table 1:** CSF analysis. AFB: acid-fast bacteria; CSF: cerebrospinal fluid; KOH: potassium hydroxide

Test	Result	Reference range
Opening pressure	13 cm H_2_O	< 20 cm H_2_0
Appearance	Clear, colorless	Clear
Glucose	63 mg/dl	50-80 mg/dl
Proteins	300 mg/dl	< 45 mg/dl
Red blood cells	250 cells/mm^3^	< 5 cells/mm^3^
White blood cells	0 cells/mm^3^	< 5 cells/mm^3^
Lymphocytes	0 cells/mm^3^	< 5 cells/mm^3^
Mononuclear cells	0 cells/mm^3^	< 5 cells/mm^3^
Lactate dehydrogenase	< 41 IU/l	< 41 IU/l
Gram stain	No leukocytes or bacteria	
KOH stain	Negative for yeast	
Ziehl-Neelsen stain	Negative for AFB	
Bacteria culture	Negative	
Fungus culture	Negative	
Mycobacteria culture	Negative	

Due to systemic symptoms, biochemical studies and autoimmunity tests were conducted, revealing high-titer positive antinuclear antibodies (ANA), consumption of complement C3 and C4, hyperlipidemia, severe hypoalbuminemia, and proteinuria. The extractable nuclear antigen (ENA) test was negative, and tests for human immunodeficiency virus (HIV), syphilis, hepatitis B, hepatitis C, and human T-cell lymphotropic virus (HTLV) infections were all negative (Table 2).

**Table 2:** Admission laboratory tests. HIV: human immunodeficiency virus; HTLV: human T-cell lymphotropic virus; LDL: low-density lipoprotein; TPPA: *Treponema pallidum* particle agglutination; VDRL: venereal disease research laboratory

Test	Result	Reference range
Leukocyte count	4.68 x 10^3^ cells/mm^3^	3.98-10.04 x 10^3^ cells/mm^3^
Neutrophil count	3.62 x 10^3^ cells/mm^3^	1.56-6.13 x 10^3^ cells/mm^3^
Lymphocyte count	0.85 x 10^3^ cells/mm^3^	1.18-3.74 x 10^3^ cells/mm^3^
Hemoglobin	12.8 g/dl	11.2-15.7 g/dl
Platelet count	368 x 10^3^ cells/mm^3^	182-369 x 10^3^ cells/mm^3^
Total serum proteins	5.24 g/dl	6.3-8.2
Albumin	1.8 g/dl	3.5-5 g/dl
Creatinine	0.6 mg/dl	0.52-1.04 mg/dl
Creatine kinase	169 IU/l	30-135 IU/l
C-reactive protein	5 mg/dl	< 9.99 mg/dl
Erythrocyte sedimentation rate	57 mm/h	1-20 mm/h
Total cholesterol	288 mg/dl	0-200 mg/dl
LDL cholesterol	146 mg/dl	< 130 mg/dl
Triglycerides	328 mg/dl	0-150 mg/dl
Complement C3	61 mg/dl	88-165 mg/dl
Complement C4	8.4 mg/dl	14-44 mg/dl
Antinuclear antibodies	1:1280 fine speckled pattern	
Extractable nuclear antibodies	Negative	Negative
Anti-DNA antibodies	Non-reactive	Non-reactive
IgM anticardiolipin antibodies	0.9 MPL-U/ml	< 10 MPL-U/ml
IgG anticardiolipin antibodies	1.6 GPL-U/ml	< 10 GPL-U/ml
IgM anti-B2 glycoprotein antibodies	0.9 IU/ml	< 5 IU/ml
IgG anti-B2 glycoprotein antibodies	2.8 IU/ml	< 5 IU/ml
Lupus anticoagulant ratio screening	0.9	< 1.2
Anti-Jo1 antibodies	1.7 IU/ml	< 15 IU/ml
HIV antibodies	Negative	Negative
VDRL	Non-reactive	Non-reactive
TPPA	0.01	< 0.08
Hepatitis B surface antigen	Negative	Negative
Hepatitis C total antibodies	Negative	Negative
HTLV I-II antibodies	Negative	Negative

**Figure 1:**
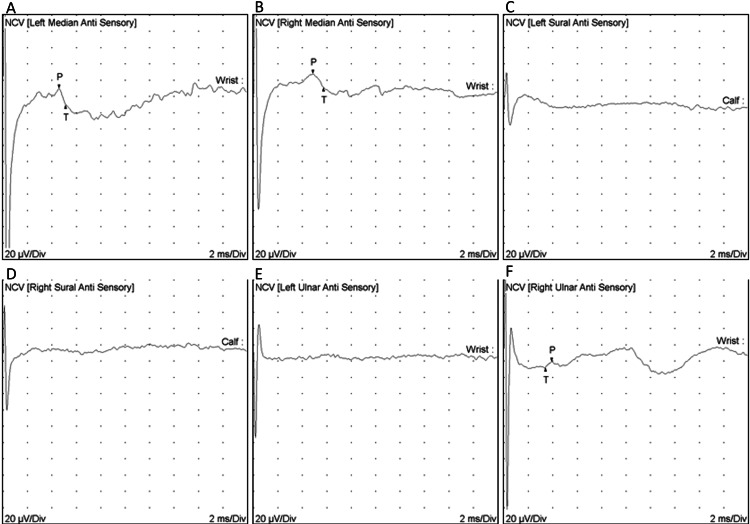
Sensory nerve conduction study. A. Left median anti-sensory. B. Right median anti-sensory. C. Left sural anti-sensory. D. Right sural anti-sensory. E. Left ulnar anti-sensory. F. Right ulnar anti-sensory. The left and right median nerves (Panels A and B), as well as the right ulnar nerve (Panel F), show prolonged distal latency and decreased conduction velocity. Both the left and right sural nerves (Panels C and D) and the left ulnar nerve (Panel E) lack excitability.

**Figure 2:**
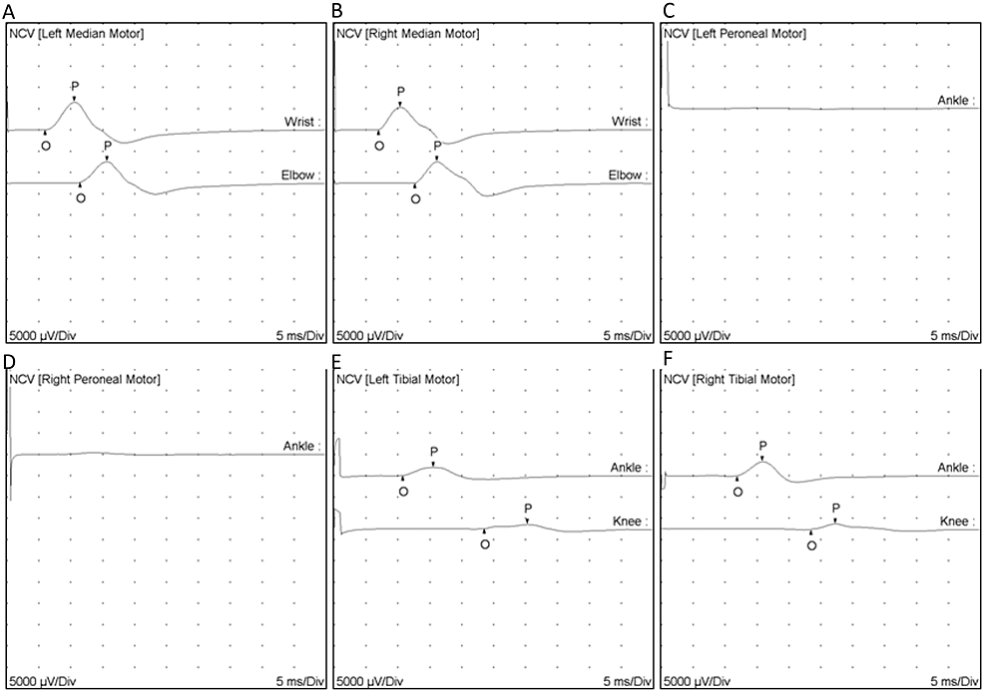
Motor nerve conduction study. A. Left median motor. B. Right median motor. C. Left peroneal. D. Right peroneal. E. Left tibial motor. F. Right tibial motor. Both the left and right median nerves (Panels A and B), along with the left and right tibial nerves (Panels E and F), exhibit prolonged distal latency and reduced conduction velocity. The left and right peroneal nerves (Panels C and D) are non-excitable.

A progressive chronic peripheral neuropathy was suspected, and nerve conduction studies of the four limbs were performed revealing a chronic demyelinating motor and sensory polyneuropathy with secondary axonal changes (Figure 1 and Figure 2).

**Figure 3:**
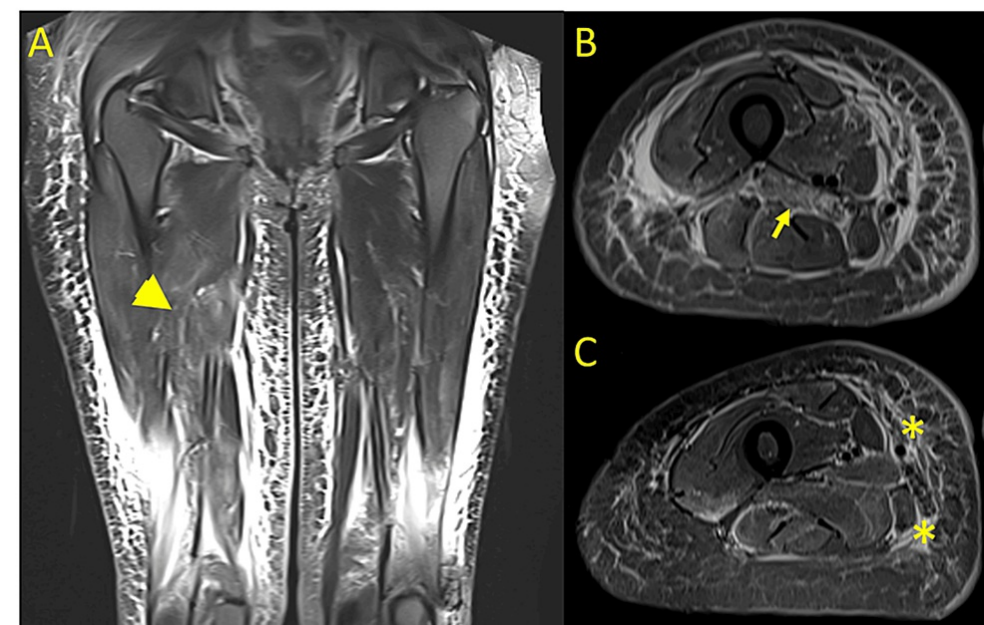
Lower limb magnetic resonance imaging. A. Contrast-enhanced T2 coronal view of both thighs. There's muscle edema predominantly in the right quadriceps (arrowhead). B. Contrast-enhanced T1 TIRM view of the left thigh showing muscle edema (arrow). C. Contrast-enhanced T2 view of the left thigh showing edema in the middle muscle compartment and subcutaneous tissue (asterisk). TIRM: turbo inversion recovery magnitude

Total creatine kinase (CK) levels were slightly elevated upon admission, so a lower limb magnetic resonance imaging was performed which showed diffuse muscle edema with predominant involvement in the quadriceps, confirming myositis (Figure 3).

A 24-hour urine collection revealed massive proteinuria of 20,235 mg, confirming nephrotic syndrome. A kidney biopsy was performed, and pathology showed a generalized increase in cellularity in the mesangial area, endocapillary proliferation, and infiltration by mixed inflammatory cells. No signs of sclerosis or structural alterations were found in the glomeruli or renal interstitium. The analyzed arterial vessels showed no abnormalities. Immunofluorescence samples revealed no identified glomeruli in the preparation, and there was no positive staining observed in tubules or vessels. The specimen was conclusive for proliferative mesangial glomerulonephritis, with the absence of immune complex deposits in the examined tissues.

Considering these findings, the diagnosis of SLE was confirmed, supported by peripheral nervous system involvement as evidenced by chronic demyelinating polyneuropathy, muscle involvement attributable to myositis, and class II lupus nephritis. Due to multisystemic involvement, particularly with renal compromise, treatment was initiated with 500 mg IV methylprednisolone pulses for three days, followed by cyclophosphamide at a dose of 750 mg/m^2^ every month for six doses, tapering prednisone, hydroxychloroquine, vitamin D, and physical therapy. Additionally, atorvastatin was initiated for secondary dyslipidemia and losartan as an anti-proteinuric measure, and warfarin was the patient's preferred choice for anticoagulation due to the risk of venous thrombosis associated with massive proteinuria and severe hypoalbuminemia.

After two months, the patient has shown significant improvement. Although she still experiences mild weakness, predominantly in the proximal lower limbs, she can walk and move independently. Serum albumin levels haven't changed, but 24-hour proteinuria improved to 6,574 mg.

## Discussion

Peripheral nervous system involvement in SLE can manifest as multiple mononeuritis, autoimmune necrotizing myopathy, autonomic neuropathy, myasthenia gravis, peripheral neuropathy, and demyelinating inflammatory polyneuropathy in the acute (AIDP) or chronic (CIDP) spectrum. These last two conditions are rare complications, occurring in as few as 0.8% of patients [1,2]. CIDP may occur simultaneously with lupus, or CIDP may precede or follow the onset of SLE [3].

CIDP is a macrophage-mediated disorder resulting from dysregulated immune response, developing over at least eight weeks. It is characterized by axonal damage and demyelination of peripheral nerves [4]. The evaluation of this condition is based on clinical manifestations, electrophysiological studies, and supportive tests such as cerebrospinal fluid analysis to confirm elevated protein levels with little to no pleocytosis [4,5]. Other diagnostic tools include magnetic resonance imaging of the cervical or lumbar plexus, ultrasound, or peripheral nerve biopsy, to differentiate between defined and possible CIDP [5].

The pathogenesis of the association between these two conditions is not clearly defined, but it is believed to involve both cellular and humoral immunity, leading to neurogenic inflammation resulting in axonal or myelin damage due to autoantibodies against nodal and extranodal structures, given the high number of antigens at the Ranvier node [6,7]. Anti-ganglioside antibodies have been described in 15-24% of patients with peripheral nervous system involvement in SLE. However, some studies did not show an association with antinuclear antibodies, anti-DNA antibodies, antiphospholipid antibodies, or low complement levels [6-9]. It is now understood that CIDP can be an autoimmune condition in its own, sharing certain HLA loci and common mechanisms with other autoimmune diseases [3].

Current guidelines for CIDP treatment recommend initiating oral or pulse corticosteroids, IVIG/subcutaneous immunoglobulin (SCIG), or plasma exchange, either alone or in combination, based on clinical response [5]. In cases of refractoriness, after assessing risks and benefits, unconventional therapies such as cyclophosphamide, rituximab, or even autologous hematopoietic stem cell transplantation may be considered [5,10]. However, the management of CIDP in patients with SLE differs from the standard approach. Conventionally, steroids have been used in combination with IVIG, as in the characterization by Julio et al., where 13 out of 16 patients received it. Nonetheless, these authors suggest that the initial treatment should be determined by the activity of SLE. In the absence of lupus activity, treatment should follow the current CIDP treatment guidelines. When lupus activity is present, corticosteroids can be combined with immunomodulators such as cyclophosphamide or rituximab. If there is no response, the initiation of IVIG/SCIG should be considered [11]. Currently, there are no clinical trials evaluating this approach, and case reports of successful treatment with immunomodulators before IVIG or plasma exchange are limited [11,12]. In the case presented here, a favorable response to initial treatment with steroids and cyclophosphamide is reported, without the addition of IVIG. This could be considered as a therapeutic alternative, especially in cases where multiple systems are involved and other manifestations like inflammatory myositis and lupus nephritis are present.

## Conclusions

This case highlights the intricate interplay between CIDP and SLE. While the exact mechanisms linking these conditions remain unclear, a tailored approach is crucial for diagnosis and treatment. Treatment decisions should consider lupus activity, and this case highlights that early intervention with steroids and cyclophosphamide, in the absence of immediate IVIG or plasma exchange, can lead to favorable outcomes. Further research is needed to establish clear management guidelines for this unique patient subset, optimizing care while mitigating the risks associated with immunomodulatory therapies.
